# Medical Fees from Hospital Patients

**Published:** 1900-06-09

**Authors:** 


### The Institutional Workshop.

### MEDICAL FEES FROM HOSPITAL PATIENTS.

One of the complications of having pay wards attached to free hospitals is that any payment calculated on the basis of the expenses incurred leaves nothing over to pay the fees of the medical officer, who, however, willing he may be to attend the poor for nothing, is by no means desirous of treating well-to-do people on the same terms. We have several times called attention to this point, and have urged that wherever patients are received who are sufficiently well off to pay for more than their bare maintenance, charges should be made according to their power to pay, rising, according to their means, to such a point that, so far as money is concerned, it should be cheaper to be treated at home, and that whenever the hospital receives from a patient more than enough to pay for maintenance a considerable proportion of the excess should go into the pocket of the doctor. Unless some such arrangement as this is made the reception of well-to-do paying patients is likely at times to lead to such regrettable incidents as that which occurred recently in Dublin, where one of the surgeons to the Mater Misericordiæ Hospital brought an action against a Mr. McHugh to recover £73 10s., being his fees for an operation and subsequent attendance upon him. The Mater Misericordiæ is a large hospital, which, in addition to receiving ordinary cases, has wards for paying patients, of whom it seems to have a considerable number, the receipts from that source having amounted to £1,180 during the year 1898. In the case to which we refer, the defendant, who had been injured in the hunting field, refused to pay anything beyond the £2 per week which he was charged by the hospital, his counsel urging that it was the surgeon's duty to attend the patients in the hospital, that the amount paid covered the attendance received, that if anyone retained the services of the surgeon it was the governors of the hospital, and that there was no contract entered into by the defendant, or any agent authorised by him, for payment of any remuneration to the plaintiff. As it seems to us this is precisely the condition of affairs in regard to most of the cases in which paying patients are received into charity hospitals, so that the result is of considerable interest. The Judge allowed the case to go to the jury, leaving to them the following questions: (1) Was the plaintiff employed by the defendant to perform the operation for remuneration? (2) If so, to what remuneration is the plaintiff entitled? The jury answered the first question in the affirmative, and the second by awarding the plaintiff £50. Stay of execution was, however, granted for a fortnight. At first sight it may seem that the simplest and the best plan for a hospital in arranging the fees it should receive from paying patients would be to separate soup from physic altogether, and to make a charge for board and lodging only, leaving all question of professional remuneration to be settled between the doctor and his patient. And if all paying patients were of such a position of life as to be able to afford full fees probably such an arrangement would be the best. But in practice it will be found that wherever there are pay wards, patients who are only very little above the position (pecuniarily) of those in the general wards will often be desirous of entering them, and for such it would be quite out of the question to charge full fees. We think, then, that on the whole the simplest plan is that an inclusive charge should be made, but that this charge should be assessed in each case, so as to be fully up to the patient's power to pay—even to the extent of covering the full fees of the doctor and of putting something into the coffers of the hospital besides. The division of the fees so received between the hospital and the medical officer could easily be made according to a sliding scale, which it would not be difficult to arrange.

